# Local Field Potential Modeling Predicts Dense Activation in Cerebellar Granule Cells Clusters under LTP and LTD Control

**DOI:** 10.1371/journal.pone.0021928

**Published:** 2011-07-19

**Authors:** Shyam Diwakar, Paola Lombardo, Sergio Solinas, Giovanni Naldi, Egidio D'Angelo

**Affiliations:** 1 Department of Physiology, University of Pavia, Pavia, Italy; 2 Consorzio Interuniversitario per le Scienze Fisiche della Materia (CNISM), Pavia, Italy; 3 Brain Connectivity Center, Fondazione Istituto Neurologico Nazionale IRCCS C. Mondino, Pavia, Italy; 4 Department of Mathematics, Milan, Italy; 5 Amrita School of Biotechnology, Amrita Vishwa Vidyapeetham (Amrita University), Kollam, Kerala, India; The Research Center of Neurobiology-Neurophysiology of Marseille, France

## Abstract

Local field-potentials (LFPs) are generated by neuronal ensembles and contain information about the activity of single neurons. Here, the LFPs of the cerebellar granular layer and their changes during long-term synaptic plasticity (LTP and LTD) were recorded in response to punctate facial stimulation in the rat in vivo. The LFP comprised a trigeminal (T) and a cortical (C) wave. T and C, which derived from independent granule cell clusters, co-varied during LTP and LTD. To extract information about the underlying cellular activities, the LFP was reconstructed using a repetitive convolution (ReConv) of the extracellular potential generated by a detailed multicompartmental model of the granule cell. The mossy fiber input patterns were determined using a Blind Source Separation (BSS) algorithm. The major component of the LFP was generated by the granule cell spike Na^+^ current, which caused a powerful sink in the axon initial segment with the source located in the soma and dendrites. Reproducing the LFP changes observed during LTP and LTD required modifications in both release probability and intrinsic excitability at the mossy fiber-granule cells relay. Synaptic plasticity and Golgi cell feed-forward inhibition proved critical for controlling the percentage of active granule cells, which was 11% in standard conditions but ranged from 3% during LTD to 21% during LTP and raised over 50% when inhibition was reduced. The emerging picture is that of independent (but neighboring) trigeminal and cortical channels, in which synaptic plasticity and feed-forward inhibition effectively regulate the number of discharging granule cells and emitted spikes generating “dense” activity clusters in the cerebellar granular layer.

## Introduction

The local field potentials (LFP) contain information about the underlying cellular activity and have extensively been used to investigate central circuit functions. In the cerebellum, the LFP evoked in the of the granular layer by mossy fiber activity was supposed to originate from granule cell spike currents, so that it could be used to gain information about the discharge of these neurons in several functional conditions [1], [2]. Micromapping experiments have suggested that granule cell activation occurs in clusters reflecting connections with different afferent fibers [3]. In response to punctate facial stimulation, the LFP is comprised of an earlier response conveyed through the trigeminal pathway (T wave) and a secondary response (C wave) conveyed through the thalamo-cortico-pontine circuit [4], [5], which could as well reflect activation of separated clusters. Interestingly the LFP can be persistently modified following patterned sensory stimulation with properties compatible with long-term synaptic plasticity (long-term potentiation and depression: LTP and LTD) at the mossy fiber – granule cell synapse [5]. However, a direct assessment of the mechanisms generating the LFP as well as of its modifications during long-term synaptic plasticity were missing.

Detailed cellular and circuit investigations have recently revealed how the granule cells respond to mossy fibers and how their output spike pattern is regulated by the number of active synapses and by the local inhibitory circuit [6], [7], [8], [9], [10], [11], [12], [13], [14], [15], [16]. The granule cells generate spike bursts limited in time by feed-forward inhibition (“time-windowing”) and in space by lateral inhibition (“center surround”) caused by the Golgi cells. Moreover, the bursts are modified during LTP and LTD at the mossy fiber – granule cell synapse through multiple expression mechanisms involving changes both in release probability and intrinsic excitability [17], [18], [19], [20]. Relevant information has also been provided about Golgi cell activation in vitro and in vivo [21], [22], [23], [24], showing short burst responses well suited for rapid and intense granule cell inhibition. These granule cells [14], [25], [26], [27], [28] and Golgi cells [29], [30] have been modeled [14], [25], [26], [27], [28] and this has allowed to integrate the various mechanisms into functional network hypotheses [31], [32], [33], [34], [35], [36], [37].

It should be possible in principle to use the extended knowledge on granule cell physiology to interpret the LFP, in particular to predict the proportion of granule cells activated in a cluster and the mechanisms through which the LFP changes during LTP and LTD. In this work we have reconstructed the LFP through a repetitive convolution of the extracellular signal generated by a detailed granule cell multi-compartmental model [26], [27], [28]. This mathematical reconstruction supports the prediction that the granular layer LFP reflects granule cell spikes, with the main current sink located in the granule cell axon initial segment. Both changes in mossy fiber - granule cell release probability and in granule cell intrinsic excitability are required to explain LTP and LTD in the LFP. The percentage of discharging granule cells in a cluster is tuned between 3% and 21% by LTP and LTD and can raise over 50% by reducing Golgi cell inhibition. These simulations allow therefore to interpret the LFP on the basis of the main cellular processes occurring in the granular layer in response to peripheral stimulation and demonstrate that LTP and LTD depend on the same blend of mechanisms revealed in vitro. The high percentage of discharging granule cells in the active clusters does not support the concept of sparseness anticipated by early theoretical models [38], [39], [40], while it is compatible with a congruent organization of granular and molecular layer activity along vertical transmission lines [41], [42], [43].

## Methods

The LFP is an ensemble signal and its interpretation involves extracting information about the constituent single neuron sources. This can be done in principle through a model containing prior information about circuit organization and about the mechanisms of extracellular current generation [44]. In this work, the LFP of the cerebellum granular layer was reconstructed through a repetitive convolution (ReConv) of the extracellular currents generated by a detailed granule cell multi-compartmental model [27]. ReConv was designed to identify salient properties of the underlying neuronal circuit on an information theoretical basis making use of a limited set of network parameters. This is indeed a novel approach. While methodological elements have been previously developed in signal theory (see below), their combination and application to LFPs generated in the brain has never been tested before. The main predictions of ReConv on network activity have been counter-tested using the available large-scale network model of the cerebellum granular layer [14] in order to independently derive the statistical parameters of network connectivity (see Appendix S1). The ReConv source codes are provided in ModelDB.

### Extracellular current and potential generated by a granule cell multicompartmental model

The two main neurons characterizing the granular layer response are the granule cells and the Golgi cells, and it is therefore important to determine their relative contribution to the LFP evoked response. The field potential [45] depends on the relative surface of the electrogenic elements of the granular layer (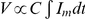, with membrane capacitance *C* being proportional to cell surface; [45]). The granule cell:Golgi cell ratio is 500∶1 for number of cells [2], [46] and 3∶50 for cell surface [18], [47]. This yields a 30 times larger electrogenic surface for granule cells, which therefore determine most of the signal (this is indeed a lower limit since some Golgi cells could be silent [21]). Thus, most of the current generating the LFP response depends on the granule cells. The Golgi cells, beside their central in determining network activity, would play a minor role in generating the LFP [10]. Conversely, current-source density analysis (CSD), which reveals oriented current flows (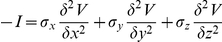, with σ being the medium conductivity along orthogonal axes in a 3D space) would be unable to reveal synaptic granule cell currents due to the random orientation of dendrites but could reveal synaptic Golgi cell currents in the apical dendrite [22].

Multi-compartmental neuron models have been successfully used to explain LFP generation in the olfactory bulb [41], [48], [49], [50] and in the hippocampus [51], [52], [53], [54]. Here we have used a detailed granule cell multi-compartmental model composed of 52 active compartments including the soma with four dendrites and an axon [27] to explain LFP generation in the cerebellar granular layer. And since all the granule cells are very similar (e.g. see [9], [55]), a single model could represent all their activities both for the T and C waves of LFP.

The model, which incorporates a detailed biophysical reconstruction of membrane mechanisms [26], [27], [28], accounts for the known electrophysiological properties of the granule cell. The Na^+^ channels include the transient, persistent and resurgent properties and are concentrated in the axon [56], [57] (a fourth property, slow inactivation, was not included [58], but it should have little relevance on the on the time-scale of these simulations). Mossy fiber - granule cell synaptic transmission was modeled using a 3-state cycle regulated by the probability of release, *p* [20], [28]. LTP and LTD were simulated by changing *p* as well as intrinsic excitability (IE) in granule cells [17]. While *p* was varied over a continuous range of values, IE was changed step-wise (increased for LTP or decreased for LTD with respect to the control state). In a previous work [28], a systematic analysis of the potential factors involved in IE changes suggested a critical role for the Na^+^ current. Here, in order to model IE changes, the sodium current steady-state inactivation curve was shifted along the voltage axis (to increase IE, C_on_ was decreased to 20% and O_on_ was decreased to 66.7% of the control value; to decrease IE, C_on_ was increased to 200% and O_on_ was increased to 133.3% of the control value – compare the kinetic scheme reported in [56], [57]).

The extracellular currents generated by the model were computed using the “extracellular mechanism” implemented in NEURON [59]. Then these currents were used to compute the extracellular potentials using a Line Source Approximation (LSA) [59] assuming a purely resistive and homogenous extracellular medium. In the case of an electrode with finite size, several compartments lay at nearly the same distance from it. Thus, the typical LFP was simply generated by summing the contribution of all somatodendritic compartments, including dendrites, soma and axon hillock (a similar reasoning applies to axonal compartments when the electrode is positioned within an axon bundle).

The extracellular potentials generated by activating the granule cell model with various synaptic combinations were used as the “kernels” for reconstructing the LFP evoked response (simply called “LFP reconstruction” throughout). With 1–4 mossy fiber synapses and 0–4 Golgi cells there are 20 independent input combinations. Following a preliminary exploration, several patterns looked very similar (redundancy). Thus, certain combinations of mossy-fiber/Golgi cell synapses (1/4, 2/3, 3/2, 4/1) were considered representative and were used for generating the *in vivo* LFP responses.

### Field potential reconstruction by repetitive convolution (ReConv)

LFP reconstruction consisted in accounting for all relevant combinations of extracellular response in a cluster of granule cells detected by the recording electrode. These responses were combined through a convolution algorithm (ReConv) taking into account the geometry and dynamics of the active granule cell cluster.

The mossy fibers were activated with short high-frequency trains (10–20 spikes/burst at 300 Hz) to mimic punctate sensory stimulation *in vivo* [6], [7], [8]. According to experimental analysis, inhibitory synapses were activated 4 ms after the mossy fibers accounting for the delays introduced by Golgi cell activation and transmission to granule cells [10], [16] (it should be noted that Golgi cells were not simulated explicitly). The proportions of active mossy fibers per granule cell was that obtained using BSS (see below). Granule cell activation was scattered using a normal distribution with 3 ms mean and 1 ms standard deviation based on experimental presynaptic neurotransmission delays [60]. Moreover, the granule cells were assumed to be homogeneously distributed in space and their contribution to the LFP was scaled depending on the distance from the recording electrode. The time delays and the different distance from the electrode cause temporal and spatial “jitter” of granule cell activity in the cluster (see below). The number of granule cells in a cluster was derived from previous works, indicating ∼600 granule cells in LFP *in vitro* [10] and ∼200 granule cells in LFP *in vivo* [5].

Mathematically, the LFP was treated as a temporal population code generated by multiple sources (signals) disturbed by jitter (noise). This case is known in signal analysis theory as “convolution of signals with noise” and uses repetitive convolution methods (ReConv) [61] that were successfully applied to SONAR and RADAR data analysis. In order to compute the LFP from multiple compartments of a single neuron model, two convolutions are performed sequentially. The first convolution averages the various possible activation patterns to generate a mean local field outside a single neuron and incorporate temporal jitter. The second convolution uses the single neuron field to estimate the LFP in the region of interest by accounting for spatial jitter caused by the localization of cells in different points of the extracellular space. The model estimates the LFP in a small region (a sphere of ∼15 µm radius), whose size was chosen by the approximate volume occupied by the cells forming the cluster. The extracellular resistivity was assumed at the standard value of 0.1 MΩ/cm (cf. [62] reporting pyramidal layer resistivity of 0.26 MΩ/cm.; see also [54]).

The ReConv algorithm consists of the following steps:

1. Sum the extracellular signals extracted from a group of compartments in neuron *i* (e.g. see Fig. 3). Repeat this operation for different mossy fiber combinations obtaining single neuron kernels, 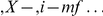.
2. Convolve the single neuron kernels, 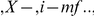 with temporal jitter, f_time_: 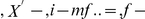

3. Weight and sum the traces 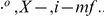 to get the prototypical filed response generated by a single neuron:
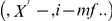

4. Iteratively, convolve using spatial jitter for the *j* ^th^ of *n* cells in the region of interest: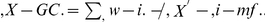. Each 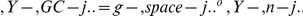 corresponds to a granule cell in the region of interest.
5. Weight and sum the traces to get the field LFP signal, 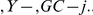

### Definition of natural activation patterns by Blind Source Separation (BSS)

Although recordings showing single mossy fiber activity have been reported [8], [23], [63], [64], [65], the distribution of mossy fiber activity to granule cells remained unknown. A Blind Source Separation (BSS) algorithm was used to estimate which proportion of granule cells, *w_i_*, is activated by a given number of fibers, 1<*n_mf_* <4 [66]. BSS was performed using JADE (Joint Approximate Diagonalization of Eigen-matrices, [67]) and allowed to identify four independent LFP components. In order to correlate the *w_i_* of each component with a specific *n_mf_*, a data mining procedure called “cross-validation” was performed [68]. In brief, the ReConv algorithm was used to generate LFPs with the computed *w_i_* and *n_mf_* values in all possible combinations. Then the LFPs were processed with CLAMPFIT in the same way as the experimental traces (see [5]). The comparison of the simulated data with the experimental waveform allowed to identify the best *w_i_* and *n_mf_* combination (2 fold cross-validation, n = 10; MSE = 0.25%; Table 1 and see Appendix S1).

**Table 1:** Cross-validation of BSS on LFPs.

*N_mf_*	*w_i_* (%) simulations	*w_i_* (%) BSS values	*w_i_* (%Error)
1	15	13.4	0.51
2	35	36.6	0.22
3	35	34.41	0.08
4	15	15.59	0.19

The table reports the relative percentage *w_i_* of granule cells receiving 1 to 4 different mossy fibers (*N_mf_*). The values used for simulation are approximations of those calculated by BSS. Note that the largest *w_i_* error occurs for single connections, which make a minor contribution to the LFP.

### *In vivo* LFP recordings

The experimental procedures adopted in this work were approved by the Ethical Committee of the University of Pavia under the protocol “Bioelectric activity in the cortico-cerebellar circuit” (9194/A of 01.08.94 renewed as 68/97-A on 23.10.97, art. 12 D.L. n.116/92) and details are fully reported in a previous paper [5]. Briefly, LFPs were recorded from Crus-IIa in urethane-anesthetized P20-P25 rats following air-puff stimulation. The 30-ms air-puffs were delivered under electronic control through a 100 µm diameter nozzle positioned about 2 mm from the skin. In order to activate different receptive fields, the nozzle was moved over the whisker pad. On each point, stimulation was repeated 50 times and the average response was taken. In some experiments, a second recording electrode was placed in the somato-sensory cortex (SI). Local cortical inactivation was obtained by pouring ice-cold saline over SI. The induction of long-term synaptic plasticity using theta-stimulation patterns followed the same paradigms reported in [5]. Usually, 10-16 consecutive traces were averaged and filtered (100 Hz high-pass, 2 kHz low-pass; the high-pass was required to stabilize the baseline avoiding slow oscillations without otherwise altering the evoked response shape). The main parameter measured in the LFP was the amplitude of the T and C waves relative to baseline (the measure was taken at peak of the two waves relative to the average value in the 100 ms preceding stimulation). Data were recorded and processed using a Multiclamp amplifier and PClamp 10 software (Molecular Devices Inc, USA).

These data were used (i) to define the aggregation in clusters of the granular layer LFP, (ii) to determine the mossy fiber input pattern, (iii) to provide the template for LFP reconstruction and (iv) to determine the LFP changes during long-term plasticity. Noisy or unstable recordings were not considered further. The recordings showing Golgi cell activity (less than 10%, characterized by large theta-frequency spikes in the granular layer), which indicates close vicinity of the electrode to a Golgi cell soma [23], [24], [69], were likewise discarded from the analysis.

### Data processing and simulation tools

Experimental and model traces were processed (filtering and averaging) using CLAMPFIT (PClamp 10, Molecular Devices Inc, USA). LFP measurements on the simulated signals were usually performed after averaging 10–16 traces and low-pass filtering at 2 kHz in agreement with the experimental case [5], [10]. The single cell model was run in NEURON [59]. All other algorithms (including those of ReConv and BSS) were developed in MATLAB (Mathworks, Gatwick, USA), which was also used for data processing.

## Results

### Cluster activation in the granular layer local field potential *in vivo*

Although the local field potentials (LFPs) elicited by mossy fiber activity in the cerebellum granular layer *in vivo* have been described in several works [1], [2], [4], [5], [70], some critical aspects remained unclear.

**Figure 1:**
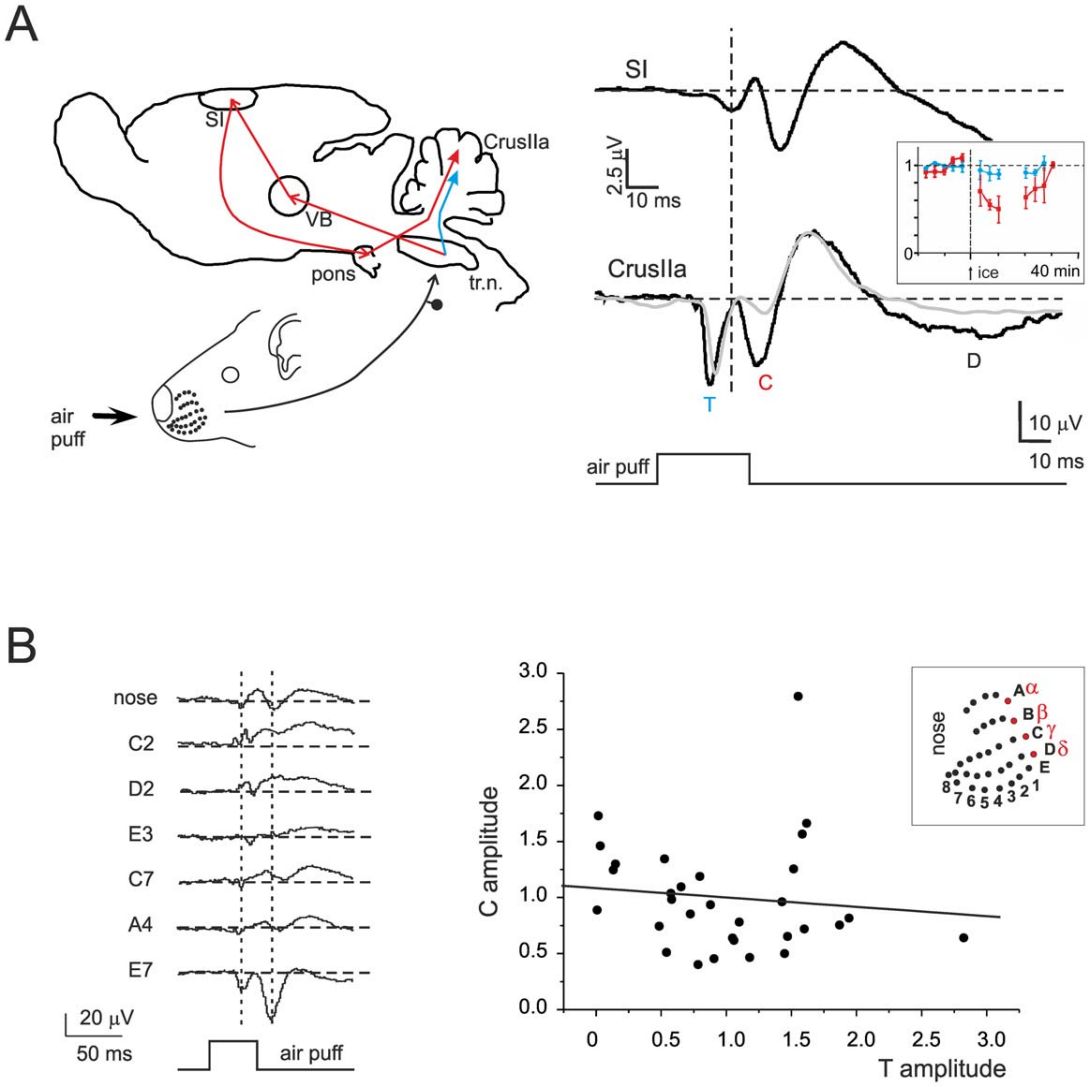
Origin of T and C waves from trigeminal and cortical pathways. (**A**) On the *left*, a schematic representation of the somatosensory circuit: tr.n. = trigeminal nucleus; VB = ventro-basal thalamus; SI = primary somatosensory cortex (controlateral); a.p. = air-puff. On the *right*, two simultaneous recordings (black traces) from SI and Crus IIa are shown: the evoked field potentials were obtained by stimulating the same whisker pad region. In Crus-IIa, T is the first to appear. Note that SI precedes C and that SI inactivation with ice-cold extracellular Krebs solution leads to a selective and reversible reduction of C but not of T (grey trace) amplitude. These observations support the model in which T derives from the direct trigeminal pathway (blue), while C has a cortical origin (red). Each trace is the average of 20 responses. The inset shows the time-course of the cooling solution. (**B**) Responses recorded from an electrode positioned in Crus-IIa granular layer and elicited by moving the air-puff stimulus in different positions (the corresponding facial coordinates of the rat whiskers are shown in the inset). The changes in wave amplitude and shape indicate different receptive fields for each location. It should be noted that, in some cases, wave polarity is inverted while changing the stimulus position. Each trace is the average of 60 responses. The plot shows the amplitude of C relative to T peak (amplitude values are normalized to the average of the responses); data are obtained from 5 different experiments with stimulation in at least 4 different whisker-pad regions. The linear fit is C = 0.08+1.08T, with R^2^ = 0.012. The T-C amplitude changes are therefore not correlated, indicating that two different clusters of granule cells are involved in generating T and C.

It has been proposed that the granular layer LFP elicited by punctate tactile stimulation is composed by two main waves: T (corresponding to direct trigeminal afferents) and C (corresponding to inputs passing through the thalamus, cerebral cortex and pontine nuclei). In a series of experiments the LFP evoked by sensory stimulation was simultaneously recorded from the somato-sensory cortex (SI) and from Crus-IIa of the cerebellum (Figure 1A). The SI response consisted of a first wave (SI_1_) arising at 18.5±0.6 ms (n = 13) and peaking at 26.9±1.1 ms (n = 13) followed by slower waves. SI_1_ anticipated C by 7.2±1.9 ms at the beginning and 6.3±1.4 ms at peak (n = 13), accounting for the time needed for spike propagation through the cortico-ponto-cerebellar pathway. SI inactivation with ice-cold saline led to a reversible reduction of C (−41.4±6.0%, p<0.05 n = 4), while T was almost unaffected (−13.8±11.5% n = 4, p = 0.18). The difference of changes in T vs. C was statistically significant, with T decreasing 3.0±0.4 times less than C (p<0.001, n = 4). Thus, C and T depended on signals conveyed by different afferent circuits and C required signal retransmission through the cerebral cortex.

A relevant issue is whether T and C waves are generated by the same or by two independent granule cell populations [4], [5]. In order to test this issue we have exploited the fact that moving the air-puff pipette over the whisker-pad allows activating different receptive fields and so different granular layer sub-fields (Figure 1B). If T and C were generated by the same granule cell population, then moving the air-puff pipette would proportionately scale their amplitude. Conversely, if different populations were involved, T and C should change independently. In fact, by changing the air-puff pipette position, T and C changed independently, so that one could increase while the other could decrease or even invert polarity. The plot of normalized C vs. T values showed points distributed around 1 with negligible slope (C = 0.08+1.08T) and a regression coefficient R^2^ = 0.012, indicating that the two parameters were statistically independent. Thus, T and C had to be generated by independent granule cell clusters.

Finally, the number of mossy fibers taking part to the excitation of individual granule cells in the local clusters is unknown. The proportion of active mossy fibers per granule cell during afferent sensory bursts was determined by BSS (see Methods) under the assumption that release probability was the same for all synapses and equal to the value measured *in vitro* (p = 0.42; [20], [71]). In 14 field potentials (taken from [5]), BSS yielded approximately 15% of 1 fiber (range 1.08 to 21.39), 35% of 2 fibers (range 14.69 to 44.91), 35% of 3 fibers (range 23.15 to 50.0), 15% of 4 fibers (range 6.89 to 22.46).

### Long-term synaptic plasticity in the granular layer local field potential *in vivo*

**Figure 2:**
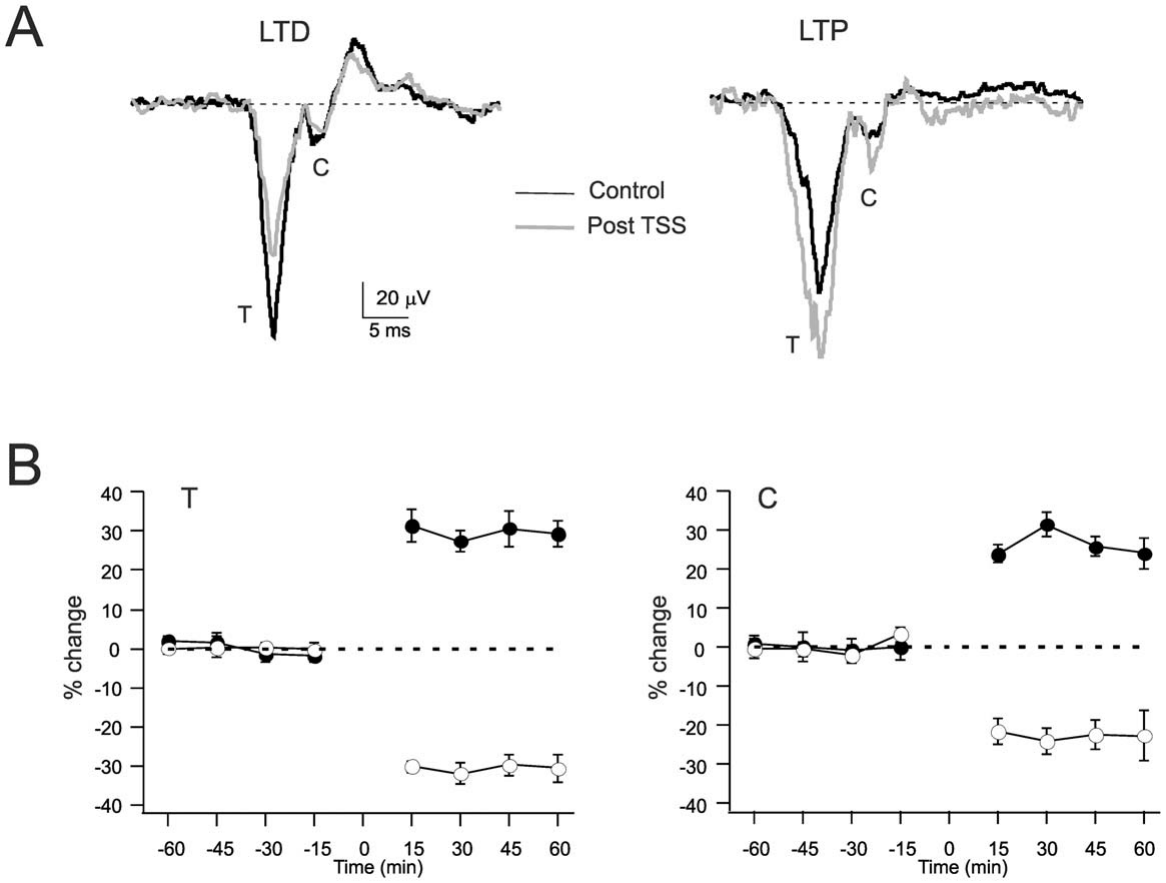
LTP and LTD of the T and C waves. (**A**) LFPs recorded from Crus IIa before (black) and after the induction of plasticity with theta-sensory stimulation (16 air puffs at 4 Hz). *Left*, recording in control. *Right*, recording in the presence of gabazine. Each trace is the average of 20 responses. (**B**) Time-course of LFP variations for the peak T and C wave amplitudes measured relative to baseline before stimulation. The data are taken either in control (open symbols) and in the presence of gabazine (filled symbols). Note that LTP and LTD are similarly expressed both in the T and C wave. Data are reported as mean ± MSE (n = 5 in all series).

It was recently shown that the granular layer LFP can be persistently modified by theta-sensory stimulation (TSS) [5]. The analysis of the T wave showed that TSS caused LTD in control, while TSS caused LTP in the presence of the GABA-A receptor antagonist, gabazine, applied to the cerebellar cortex. Here, the analysis of TSS effects was extended to the C-wave either in control conditions (n = 5) or during perfusion of the GABA-A receptor antagonist, gabazine (TSS+gabazine, n = 5). Both T and C showed LTD in control and LTP with gabazine (Figure 2), so the two waves co-varied. The T and C wave changes (both measured relative to baseline) are reported in Table 2.

**Table 2:** Field potential changes after TSS.

	TSS		TSS with gabazine	
	T	C	T	C
**Experiments**	−33.9±8.9n = 5p<0.01	−25.2±7.0n = 5p<0.01	24.2±5.2n = 6p<0.005	18.7±6.0n = 4p<0.05
**Model**	−13.9	−41%	18.21	34.9

The field potential changes after TSS were recorded either in control conditions or with gabazine superfusion (t = 30 minutes after induction). Both T and C co-vary during LTD and LTP with gabazine.

### Local field potential reconstruction by repetitive convolution techniques

**Figure 3:**
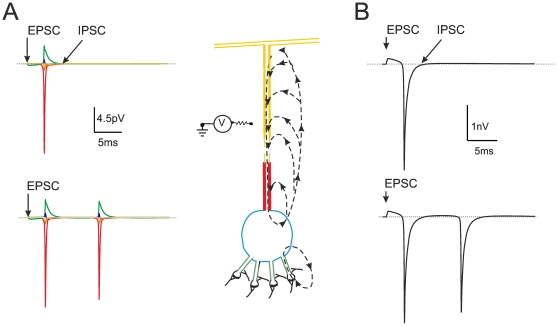
Extracellular field potential generated by a single granule cell. (**A**) Schematic representation of a granule cell according to the model of Diwakar et al. [27]. The granule cell generates synaptic responses in the dendritic endings and action potentials in the axon hillock. This forms two current sinks, with the axon hillock giving by far the major contribution. The broken arrows depict the current flow, colors indicate the major neuronal comportments. The Na^+^ channels are concentrated in axon hillock, as indicated by immunohistochemistry [43], the excitatory and inhibitory synaptic channels are located in the terminal dendritic compartments. The circuit schematics on the left shows the flow of transmembrane current over the extracellular resistance. The extracellular potentials generated by different compartments of the granule cell model are shown to the left (same colors as in the neuron compartments). Notice that the extracellular potential is the largest in correspondence of the hillock, where Na^+^ channels have the highest density. (**B**) Extracellular field potential generated by a single granule cell “seen” from an electrode covering soma, dendrites and axon hillock (corresponding to a granular layer sink). Both in A and B, the neuron responds to the synchronous activation of all four mossy fibers (and all four inhibitory synapses, when active). Both in A and B, the neuron generates a single spike when synaptic inhibition is active, while it generates a doublet when synaptic inhibition is turned off.

The hypothesis on the generation of the local field potential of the granular layer dates back to the original observations of Eccles et al. [1], [2], who proposed that the LFP was determined by granule cell discharge with the spike current sink in the granular layer and current source in their axons (the parallel fibers). Accordingly, the polarity of the LFP reversed when the electrode was moved from the granular to molecular layer. The mechanism generating the LFP was reconstructed here by using a granule cell multi-compartmental model (Figure 3A–B). These simulations showed that spikes made the largest contribution to the field potential with the major current sink in the axon hillock where Na^+^ channels are concentrated [56]. Synaptic currents gave a smaller contribution with the sink in the dendritic tip. This arrangement differentiates granule cells from neurons like pyramidal cells, in which the extended dendritic tree endowed with synapses and Na^+^ channels causes a prevalent sink in the dendrites [31], [72].

**Figure 4:**
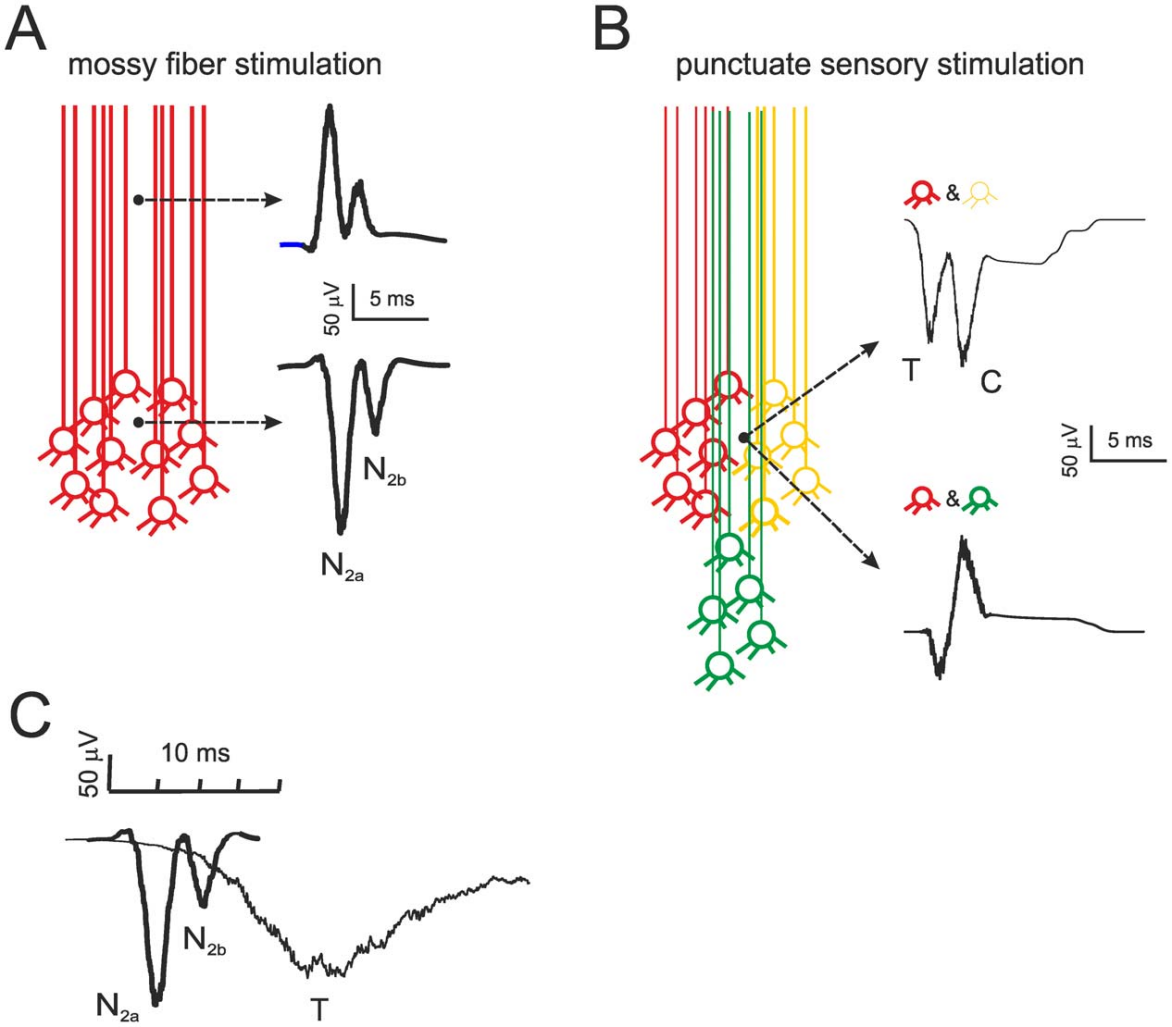
Mathematical reconstruction of local field potentials by repetitive convolution. (**A**) LFP simulating *in vitro* recordings in response to a single stimulus. The N_2a_ and N_2b_ waves are generated by a single cluster composed of 600 granule cells and are caused by spike doublets in granule cells in the absence of inhibition. The shape of the waveforms is expression of the temporal and spatial jittered convolution of several individual responses (cf. Figure 3). (**B**) LFP simulating *in vivo* recordings in response to a train of 3 stimuli at 300 Hz. The T and C waves are generated by different granule cell clusters with inhibition arriving 5 ms after the beginning of the stimulus. When both clusters surround the electrode, the two waves show negative polarity. However, C is inverted once the activated cluster moves proximally and the electrode records from axons. (**C**) The different time-scale of the response to a single stimulus and to bursts of stimuli is shown for comparison.

In order to reconstruct the LFP, the mossy fibers were activated using either a single pulse to mimic electrical stimulation for *in vitro* simulations or short high-frequency trains mimicking punctate sensory stimulation for *in vivo* simulations (Figure 4) [6], [7], [8]. The proportions of mossy fiber – granule cell connections were identified by BSS (Table 2). Golgi cell inhibition was assumed to occur synchronously after 4 ms on all the granule cells [10], [16], [73], [74]. The LFP was then reconstructed by convolving granule cell responses evoked by mossy fiber activity in time and space through jitter functions (ReConv, see Methods). Since the granule cells emit ascending axons bifurcating into the parallel fibers as they reach the molecular layer, an electrode located into a granule cell cluster would detect the signal from the spike current sink, while an electrode located into the axon bundle would be recording from the source [2]. Accordingly, the sign of the simulated field potential was inverted in the two cases explaining the experimental observations (Figure 4). In particular *in vivo,* by moving the stimulation point on the whisker pad, the T and C waves recorded from the granular layer change disproportionately and can even assume opposite sign. This could be easily explained if T and C, which depend on the trigeminal and cortical input, were generated by different granule cell clusters contributing either to sink or source signals (cf. Figure 1B).

### Modeling the LFP response to single mossy fiber pulses: *in vitro* case

**Figure 5:**
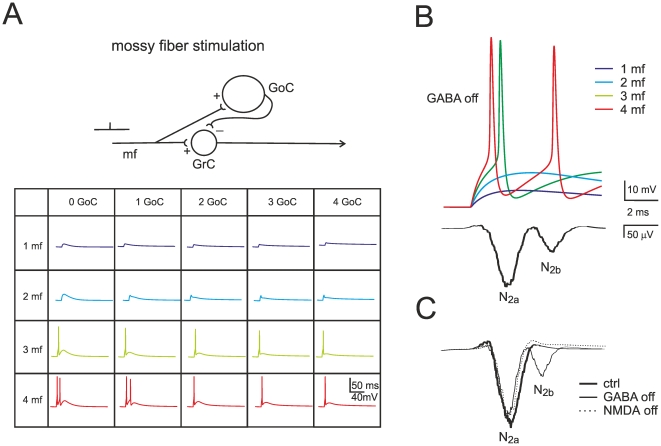
Mechanism of local field potential generation *in vitro*. (**A**) Granule cell (GrC) responses generated by combining the input from 1 to 4 mossy fibers with inhibition from 0 to 4 Golgi cells (GoC). This gives rise to 20 fundamental combinations. Excitation and inhibition consist of a single pulse, with inhibition occurring 4 ms after the beginning of excitation. N_2a_ is generated by the first and N_2b_ by the second spike in a doublet. Schematics of the circuit are shown at the top. (**B**) The LFP *in vitro* is generated by jittered convolution of different responses in a 600 granule cell cluster, four of which are shown at the top. (**C**) The LFP control by synaptic receptors accounts for experimental observations: N_2b_, but not N_2a_, is increased by GABA-A receptor switch-off and reduced by subsequent NMDA receptor switch-off.

In the circuit used for field potential reconstruction *in vitro* (Figure 5A), the mossy fibers were activated with a single pulse and the granule cells composed a single homogeneous cluster. Depending on the different combinations of active mossy fibers and granule cells, different responses could be obtained (Figure 5A, inset). The proportion of active mossy fibers per granule cell was determined by using BSS from 20 LFP recordings from brain slices yielding, approximately: 5% 1 fiber (range 1.99 to 9.08), 45% 2 fibers (range 37.70 to 50.0), 35% 3 fibers (range 26.14 to 45.50), 15% 4 fibers (range 7.21 to 20.21). With these parameters and 700 granule cells in the cluster, the model reproduced the field potential measured *in vitro* showing an appropriate proportion between N_2a_ and N_2b_ waves (Figure 5B).

In addition, the model generated three major predictions (Figure 5B–C). First, spike doublets in granule cells were the cause of N_2a_ and N_2b_ in the LFP. Secondly, synaptic inhibition, because of its delayed activation, controlled generation of the second spike in the doublet thereby regulating N_2b_ amplitude. Thirdly, the NMDA component, because of its slow raise, was the main responsible for generating the second spike and N_2b_. Collision of NMDA receptor-mediated depolarization with Golgi cell inhibition regulated doublet generation. The model captured, therefore, the main mechanistic properties of LFPs revealed by simultaneous multi-electrode array, patch-clamp and pharmacological analysis in acute cerebellar slices [15].

### Modeling the LFP response to single mossy fiber pulses: *in vivo* case

**Figure 6:**
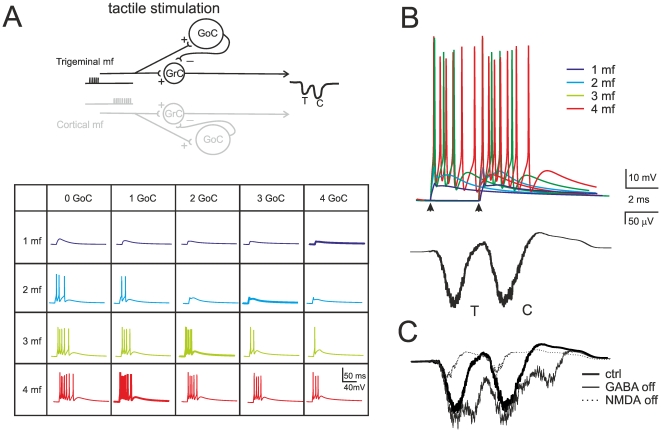
Mechanism of local field potential generation *in vivo*. (**A**) Different granule cell responses generated by combining the input from 1 to 4 mossy fibers with inhibition from 0 to 4 Golgi cells (GoC). Excitation consists of a pulse train at 500 Hz and inhibition of 1 impulse occurring 4 ms after the beginning of excitation. Due to redundancy, 4 out of 20 patterns (GrC/GrC = 1/4, 2/3, 3/2, 4/1; thicker lines) are sufficient to adequately reconstruct the LFP. The T and C waves are generated independently (C has a delay of 10 ms) and then summed linearly. Schematics of the circuit are shown at the top, illustrating independent circuits for T and C. (**B**) The LFP *in vitro* is generated by different combinations of responses, four of which are shown at the top. (**C**) The LFP control by synaptic receptors accounts for experimental observations: T and C are increased by GABA-A receptor switch-off and reduced by subsequent NMDA receptor switch-off.

*In vivo* field potentials were reconstructed by activating the mossy fibers with short spike bursts generating EPSPs and spikes in granule cells [6], [7], [8]. T and C waves were generated independently using 500 Hz trains, with C delayed by 10 ms to respect the timing observed *in vivo*. The inhibition coming from Golgi cells arrived with 4 ms delay and consisted of a short IPSP burst (3 IPSPs at 100 Hz) in the granule cell (Figure 6A; [23], [29], [30]). By using the mossy fiber proportions reported above and 220 granule cells in the cluster, ReConv generated T and C waveforms with appropriate timing and shape (Figure 6B). The simulations were carried out with independent inhibition over the two granule cell populations.

The simulated field recordings showed the typical pharmacological changes observed experimentally (Figure 6C; cf. [5]). Blocking GABA-A receptors increased and protracted the T and C waves and raised the proportion of cell making spikes from 12.5% to 50.1%. Blocking NMDA receptors strongly reduced the waves almost abolishing the ability of making spikes ([9]; see also the recent demonstration in NR2A knock-out mice, [75]), and no further changes were determined by subsequent blockage of GABA-A receptors [10]. These results demonstrate that the reconstruction of granular layer field potentials can capture the main properties of the underlying cellular mechanisms, estimating at the same time the proportion of active granule cells in responding clusters *in vivo*.

### Prediction of the effects of long-term synaptic plasticity on the LFP *in vivo*

**Figure 7:**
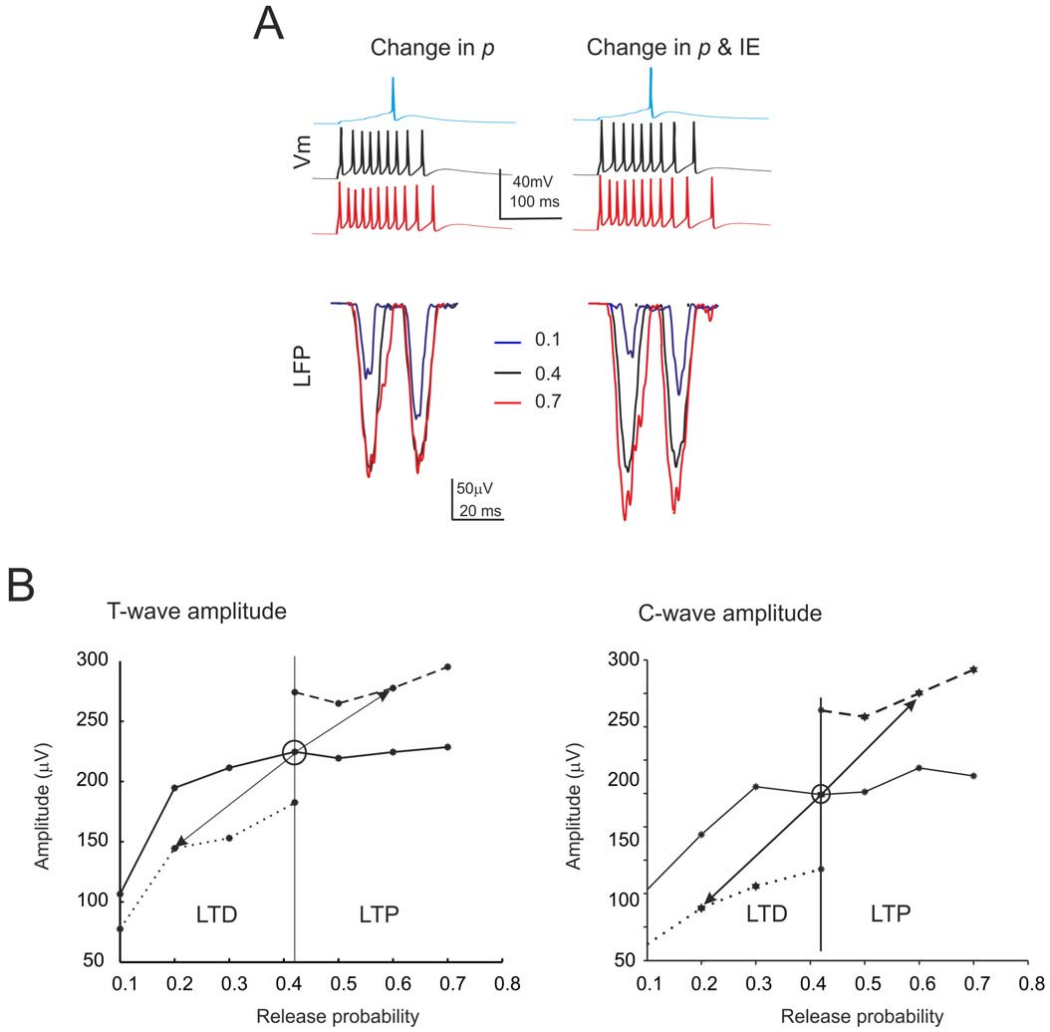
Simulations of the effects of long-term synaptic plasticity -I. (**A**) Simulated intracellular changes (top) and LFP changes (bottom) *in vivo* obtained by changing release probability (*p*) alone (left) or together intrinsic excitability (IE) (right). (**B**) The plots show the LFP T-wave and C-wave peak amplitude changes caused by release probability, *p*. The three different curves represent levels of IE (low, normal and high). Note that LTP and LTD changes similar to those observed experimentally occur when both *p* and IE change bidirectionally around the control value. Each trace is the average of 15 simulations.

The LFP undergoes plastic changes *in vivo* in the form of LTP and LTD [5]. In order to simulate the impact of long-term synaptic plasticity on the LFP, we have systematically changed release probability (*p*), which has been reported to increase during LTP and to decrease during LTD *in vitro* [15], [19], [20]. As the natural distribution of *p* values is unknown, we have assumed that the *p* value is the same for all the fibers. Since the T wave is of easier interpretation [5], simulations were first carried out to investigate the effect of *p* on the T wave (Figure 7, Table 2).

In these simulations, T changed with *p* but the T amplitude was highly sensitive to *p* changes only for *p* values lower than 0.4. Thus, the LFP plasticity curve based on *p* changes was asymmetric. This raises an issue on what the LFP can reveal about plasticity, since LTP and LTD are nearly symmetric and bidirectional in nature [5], [19]. One possibility is that the average resting *p* value is lower *in vivo* than *in vitro*. Alternatively, LTP may occur mostly at synapses with low initial *p* and LTD at synapses with high initial *p* [20]. Yet another possibility is that *p* changes are not sufficient to explain the long-term synaptic plasticity of LFPs *in vivo*. It has been reported that changes in granule cell intrinsic excitability (IE) usually occur during LTP *in vitro* and enhance spike generation [17]. We have therefore simulated a bidirectional change in IE, as reported in hippocampal neurons [76]. The excitability of granule cells was modulated by changing the activation and inactivation time constants of the sodium current (see Methods). By combining pre- and postsynaptic changes, amplitude sensitivity was extended symmetrically over the whole *p* range (Figure 7) so that, by moving from the resting state (*p* = 0.4) by 0.2 *p* units allowed to obtain an LFP amplitude change comparable to LTP and LTD measured experimentally on the T wave ([5]; cf. Table 2).

The simulation were then used to analyze the C wave changes during plasticity. When the model was used to simulate the effect of TSS in control condition (LTD with decreased *p* and E/I), the C amplitude decreased. When the model was used to simulate the effect of TSS with inhibition blocked (LTP with increased *p* and E/I), the C amplitude increased. Therefore, the model predicted that the C wave changes had always to co-vary with the T wave, as indeed observed in recordings *in vivo* (cf. Figure 2 and Table 2).

**Figure 8:**
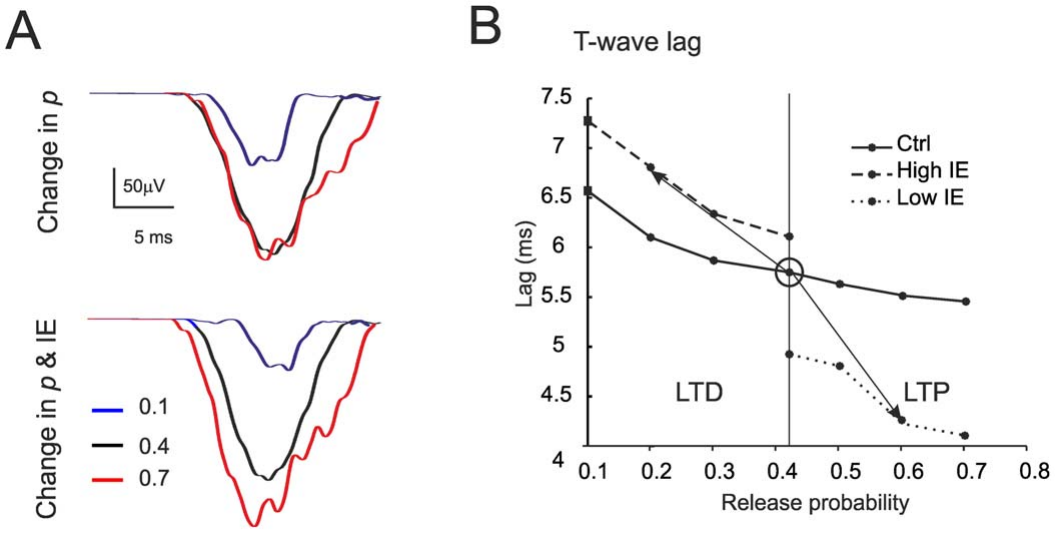
Simulations of the effects of long-term synaptic plasticity -II. (**A**) Simulated LFP changes *in vivo* obtained by changing release probability (*p*) alone (top) or together intrinsic excitability (IE) (bottom). The rising phase of the LFP is shown on expanded time-scale. (**B**) The plot shows the delay changes caused by *p* in T-wave. The three different curves represent levels of IE (low, normal and high). Note that appropriate LTP and LTD changes like those observed experimentally occur when both *p* and IE change bidirectionally around the control value. Each trace is the average of 15 simulations.

The other parameter sensitive to LTP and LTD is the lag to the T wave. This lag is related to the spike delay shift caused by changes in *p* and the consequent modification of the temporal summation rate of EPSPs [28]. The lag was most sensitive to *p* changes at low *p* and its sensitivity was extend over the whole *p* range following changes in IE (Figure 8).

In the simulations, LTP and LTD were accompanied by changes in the proportion of discharging granule cells. In naïve conditions (*p* = 0.4), following burst activation, spikes arose in 12.5% of the cells and the proportion became 21.25% with LTP and 3.75% with LTD. Thus, the proportion of discharging granule cells decreased or increased without either going to zero or saturating.

## Discussion

This paper explains the generation mechanisms of local field potentials (LFPs) in the cerebellar granular layer and predicts the LFP changes caused by LTP and LTD. The T (trigeminal) and C (cortical) waves of the LFP *in vivo* reflected independent granule cell clusters, whose activity was reconstructed using a mathematical simulation. LFPs reconstruction was based on repetitive convolution (ReConv) techniques applied to extracellular currents generated by a detailed multi-compartmental model of the granule cell [27]. Simulations predicted that LFP changes occurring during LTP and LTD had to be sustained by simultaneous changes in both mossy fiber release probability (*p*) and granule cell intrinsic excitability [28]. The same mechanism could be applied to both the T and C waves of the LFP, suggesting that plasticity occurs in different clusters with similar mechanisms independent from the origin of afferent fibers. In the active granule cell clusters, the percentage of granule cells emitting spikes in response to a punctate somato-sensory stimulus was ∼11% and, depending on whether LTD or LTP were expressed, this values ranged from 3% to 21%. However, in the absence of synaptic inhibition, the same patterns activated more than 50% of the granule cells. These simulations suggest therefore that, during natural stimulation, long-term synaptic plasticity and feed-forward inhibition regulate the number of active granule cells generating “condensed” activity clusters in the cerebellum granular layer [33].

### Reconstruction of the granular layer local field potential

The simulations allowed to reconstructing the granular layer LFP both *in vitro* and *in vivo.* During spikes, the granule cell axon hillock acted as a powerful sink of Na^+^ current while the rest of the neuron acted as a passive source [27], providing most of the extracellular current flow generating the LFP. Our simulations thus support the original LFP interpretation put forward more than 50 years ago [1], [2]. In granule cells, the maximum synaptic current is about 10 times smaller than the spike current [20], [26], [56], so that the spike gives the major contribution to the LFP. This situation is opposite to that observed in neurons of the olfactory bulb [41], [48], [49], [50] and cerebral cortex and hippocampus [51], [52], [53], [54], in which the contribution of synaptic currents to the LFP is prevalent.

ReConv assumes that (i) several granule cell discharge patterns are redundant and cannot be tied apart (i.e. a limited set of patterns is representative for all possible patterns), that (ii) granule cells are similar one to each other, that (iii) connectivity is the same in the whole cluster, that (iv) LTP and LTD are homogeneously distributed in the cluster, and that (v) the extracellular matrix of the cluster is isotropic. BSS assumes that the number of active mossy fibers per granule cells is the main determinant of response pattern generation in the LFP. The marked adherence of simulations to experimental results suggests that the assumptions used for ReConv and BSS are not critical to interpret the LFP, and this conclusion is fully supported by the large-scale model simulations reported in Appendix S1. This implies that the ReConv and BSS provides an effective “mean-field” approximation of granular layer cluster activity. This probably reflects the fact that granule cells have indeed a stereotyped firing pattern and tend to discharge in a restricted time window [9], [55], [77], [78] due to the almost synchronous feed-forward inhibition through Golgi cells, which is enforced by network connections and gap-junctions [10], [16], [73], [74]. Moreover, the extracellular matrix has a nearly-random organization characterized by dendrites and axons traveling in various directions (e.g. see histological preparations in [1], [2], [44]).

### Prediction of activity in the granular layer clusters

The results reported in Figure 1 and in subsequent simulations indicate that, in the granular layer circuit, specific receptive fields activate granule cells clusters segregated (but not far) from those receiving the corresponding signals coming from cerebro-cortical loops. It is thus possible that these clusters are synchronized through a common Golgi cell inhibitory network [73], [74] and project to the same (or to the same group of) Purkinje cells [35]. Indeed simulation results were similar when a common inhibitory pattern was used to inhibit both granule cell populations (data not shown).

According to single cell recordings *in vitro* and *in vivo*, in the simulations the granule cells responded with short spike bursts to bursts in the mossy fibers [6], [8] and the Golgi cells inhibited the granule cells in 3–5 ms [10], [16]. This mechanism, which limits the number of output spikes [33], effectively determined the amplitude and width of the T and C waves of the LFP. Thus the LFP is sensitive to the number of spike and to the temporal pattern of granule cell discharge.

By using the number of cell in a cluster estimated from morphological and imaging data (600–700 granule cells: [12], [79], [80]) and a standard value for extracellular resistivity (0.1 MOhm/cm) [54], [62], the LFP was 2–3 times larger than observed experimentally (e.g. cf. Figure 4 to Figure 1). Thus, the extracellular resistivity of the granular layer may be overestimated and a realistic value may approach that reported for the cortical pyramidal layer (0.26 MOhm/cm [54]). With 600–700 granule cells and a proportion of activation of 11%, about 60–70 granule cells in a cluster would emit spikes following a punctate sensory input. Due to the center-surround effect caused by lateral inhibition [10], most of the active granule cells should be located into the core. This conclusion is supported by simulations using a large-scale granular layer model [14] (see Appendix S1) showing that the probability of firing increases from an average value of 11% to 30% in the core. Therefore, when a cluster is activated, firing should involve a high percentage of active granule cells (actually 180–200) all condensed into the core.

### Prediction of composite mechanisms of LTP and LTD *in vivo*

These simulations show that the expression mechanisms reveled *in vitro* can explain the LFP changes associated with LTP and LTD *in vivo* [5]. The amplitude and delay of the simulated T-wave were very sensitive to *p* changes [19], [20] at low *p* but saturated at higher *p* values typical of LTP. The proper sensitivity was restored by raising intrinsic excitability during LTP [17], in agreement with the fact that the LFP mostly reflects the number of spikes generated by granule cells. As a corollary, intrinsic excitability may be reduced during LTD (as observed in hippocampal neurons [76], [81]), although this hypothesis awaits for experimental confirmation and is not strictly necessary to explain the results of simulations. Alternatively (or in addition), *p* may be lower *in vivo* than *in vitro* and LTP could occur more easily at synapses with low initial *p* [20], bringing the LFP changes into the high sensitivity region of the plasticity curve shown in Figure 7.

By using the same blend of plasticity mechanisms, simulations predicted changes in the C-wave similar to those in the T-wave. Although it cannot be excluded that additional plasticity may arise before the cerebellum in the thalamo-cortico-pontine circuit or that changes may occur the in the T-C through (which reflects inhibitor process and spike AHP), these observations suggest that the same mechanisms of induction and expression apply to mossy fiber – granule cell synapses along both trigeminal and cortical afferent pathways.

### Conclusion

The emerging picture is that independent trigeminal and cortical channels converge toward neighboring granular layer regions of the cerebellum and, despite their different origin, make use of similar mechanisms of synaptic excitation and plasticity [41]. The high percentage of discharging granule cells in the active clusters suggests that information transmitted through these channels is “dense” rather than “sparse” (meaning that just a limited number of neurons respond to the input, say <1%, [39]: the sparseness hypothesis was originally put forward in the Motor Learning Theory to explain the expansion recoding process and was through to be as a pre-requisite for efficient learning at the parallel fiber – Purkinje cell synapse [38], [40]). Multiple active clusters, by causing the congruent activation of the overlaying Purkinje cells [41], [43], are expected to determine the dynamic “spot-like” activity of Purkinje cells observed *in vivo* [42]. Moreover, while Marr predicted that plasticity at the mossy fiber – granule cell relay would “sooner or later saturate” being therefore computationally irrelevant, these simulations suggest that saturation is never attained in the cluster. Rather, long-term synaptic plasticity together with synaptic inhibition, by controlling the proportion of discharging granule cells and the number of emitted spikes, could fine tune the delay and gain of transmission in the clusters [33]. The experimental testing of these predictions will require further electrophysiological and imaging investigations of granular layer activity and computational modeling of the cerebellum and of the cerebro-cerebellar control loops [39].

## Supporting Information

Appendix S1 Supplemental Material.
(DOCX)
