# Supplementary material for: Local Field Potential Modeling Predicts Dense Activation in Cerebellar Granule Cells Clusters under LTP and LTD Control
Source: PLoS One. 2011 Jul 19;6(7):e21928. doi: 10.1371/journal.pone.0021928 (PMC3139583; doi:10.1371/journal.pone.0021928)
Supplement: Appendix S1 — Supplemental Material. (DOCX) [file pone.0021928.s001.docx]

**Supplemental Material**

Predictions of granule cell activity by local field potential modeling: conditions for plasticity

Shyam Diwakar ^1,2,5^, Paola Lombardo^1^, Sergio Solinas^3^, Giovanni Naldi ^4^, Egidio D’Angelo^1,3^

^1^Department of Physiology, University of Pavia, Via Forlanini 6, I-27100, Pavia, Italy.

^2^Consorzio Interuniversitario per le Scienze Fisiche della Materia (CNISM), Via Bassi 6, I-27100 Pavia, Italy

^3^Brain Connectivity Center, Istituto Neurologico IRCCS Fondazione C. Mondino, Via Mondino 2, I-27100 Pavia, Italy

^4^Department of Mathematics, University of Milan, 20133, Milan, Italy.

^5^Amrita School of Biotechnology, Amrita Vishwa Vidyapeetham (Amrita University), Kollam, Kerala, 690525, India.

**Neuronal activity in a large-scale model of the granular layer**

The results obtained with ReConv have been tested using a detailed large-scale model of the granular layer including about 10^5^ granule cells and tens of Golgi cells [1]. This model explicitly represents all neurons and their synaptic connections. Thus, it can provide indications about the granule cell discharges and the percentage and distribution of active cells in a cluster validating the assumptions used in ReConv. However, the large scale model is computationally intensive (tens of times slower than ReConv) and does not include multicompartmental granule cells models or mechanisms for local field potential reconstruction.

The large scale model used for these simulations is identical to that published previously, except for the fact that the number of granule cells was increased to 100000 with 1000 Golgi cells/granule cell in order to have a more precise representation of cell proportions. Moreover, mossy fibers were branched generating a cluster of rosettes in a 200x200x30 μm volume in the sagittal plane [4]. Finally, synapses between mossy fibers and Golgi cell have been increased from 50 to 150. This allowed to accelerate the rate of Golgi cell synaptic depolarization when a small mossy fiber bundle is activated, improving control over the timing of inhibition [2,3] (Appendix S1 Figure1). With 23 active mossy fibers, the granule cell cluster included about 650 granule cells and inhibition in granule cells peaked in about 4 ms. Therefore, this cluster is similar to those observed experimentally [2,4,5,6,7,8,9] and reconstructed by ReConv. The proportion of active mossy fibers per granule cells also matched the BSS results reported in the main paper (Appendix S1 Figure 2).

The size of a cluster is determined by the number of the granule cells receiving at least one active synaptic input from the mossy fibers. In large-scale simulations, a cluster was generated by activating a bundle of mossy fibers (Appendix S1 Figure 3A). While the number of granule cells increased linearly with the number of mossy fibers (from 6 to 23), the percentage of granule cells emitting spikes (or active granule cells) remained similar. With a standard release probability value in all the mossy fiber – granule cell synapses of the circuit (p=0.42), the percentage of active granule cells was around 11%, yielding a value almost identical to that obtained with ReConv.

To simulate LTP and LTD, the value of *p* was increased or decreased homogeneously in the whole cluster. In these conditions, the percentage of active granule cells decreased to around 5% with LTD (p=0.2) and increased to around 20% with LTP (p=0.8). The LTP and LTD results (Appendix S1 Figure 3A) are thus consistent with ReConv. The slightly smaller change observed here probably reflects the lack of intrinsic excitability changes. These simulations show that the proportion of discharging granule cells decreased from center (where it was as high as about 50%) to periphery of the active area, in agreement with the “center-surround” mechanism [5] (Appendix S1 Figure 3B). Following a *p* change, the density distribution of active cells did not change significantly. The increase in *p* was also accompanied by an anticipation of the first spike and by an increase in the number of spikes per cell (Appendix S1 Figure 2C), in agreement with the “time-window” mechanisms [8].

A further extension of the model could derive from implementing learning rules, which can isolate an area making LTP in the core from another making LTD in the surround. Recently, the this same large-scale computational model of the granular layer has been adapted to generate long-term synaptic plasticity in response to afferent mossy fiber bursts. A simple learning rule was elaborated in order to link the average granule cell depolarization to LTP and LTD. Briefly, LTP was generated for membrane potentials >-40 mV and LTD for membrane potentials <-40 mV. The result was to generate LTP and stronger excitation in the core of active clusters, which were surrounded by LTD. These changes were accompanied by a faster and stronger spike generation compared to the surround. However, the profile of the discharging area obtained at p=0.8 was very similar to that observed applying the voltage-dependent plasticity rule, yielding 22% discharging cells [10]. Therefore, the differential localization of LTP and LTD in the center and in the surround does not substantially alter the average proportion of discharging granule cells in the active cluster.

The results reported in Appendix S1 Figures 2 and 3 show that the response properties were rather insensitive to the number of mossy fibers as far as the clusters were between 200 and 650 granule cells. Moreover, they show that the center-surround structure and the segregation of LTP and LTD in this center-surround do not influence remarkably the LFP interpretation based on ReConv. In aggregate, the simulations carried out using the large scale model suggest that the assumption used in ReConv were not critical and that ReConv provides a valid “mean-field” approximation of the LFP.

**Appendix S1 Figure 1. The impact of inhibition in the large-scale model. (A)** The time delay for the generation of inhibitory responses in granule cells decreased with the number of active mossy fibers. This reflected the fact that Golgi cells depolarization (and therefore spike generation and subsequent GABA release) is accelerated when a higher number of mossy fiber synapses is activated. **(B)** Inhibitory conductances in the active (i.e. spiking) granule cells in a cluster activated by 23 mossy fibers. Note the presence of background activity and the sudden raise in inhibitory conductances about 4 ms after mossy fiber stimulation (arrow). The red trace is the average inhibition in the whole granule cells set.

**
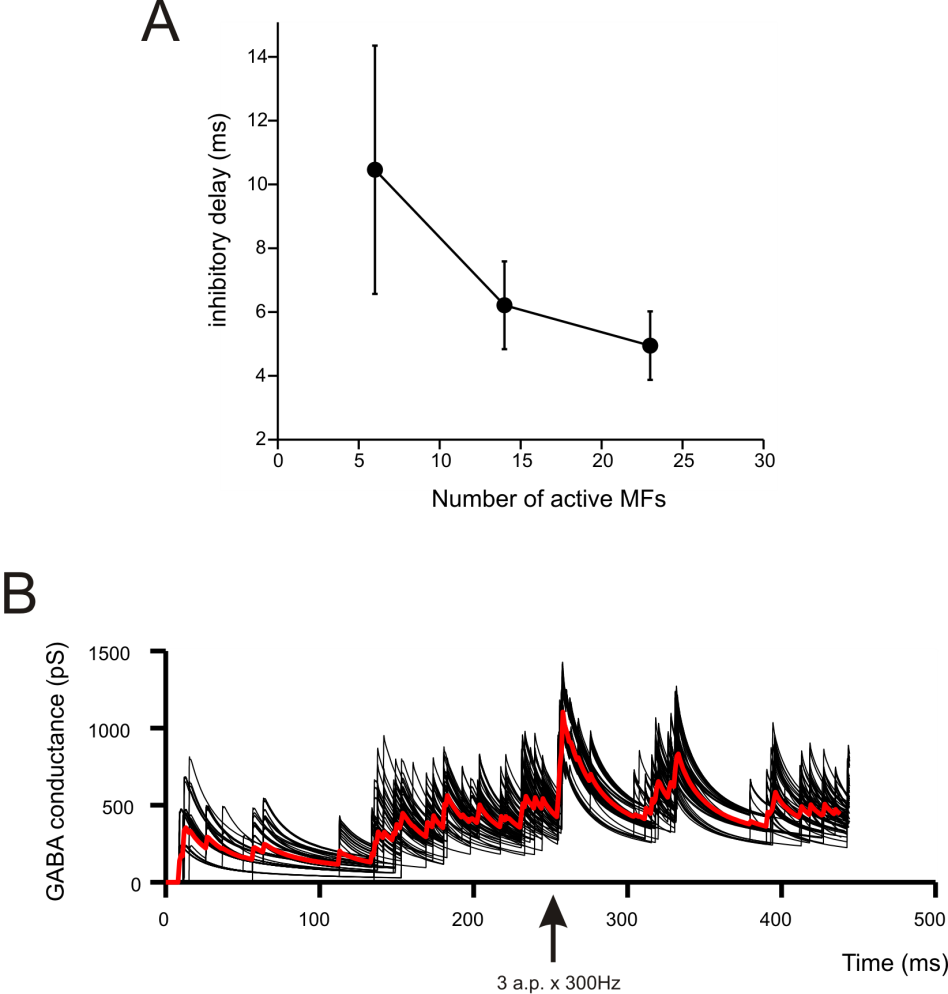
**

**Appendix S1 Figure 2. Proportion of active mossy fibers per granule cell.** The proportion of granule cells activated by 1 to 5 mossy fibers is reported from the large scale model (gray tones) and from BSS measurements (blue tones). The calculation using the large-scale model were carried out by considering the granule cells activated by a bundle of mossy fibers, in which excitation overcame inhibition. These granule cells are those contributing the most to the ensemble response similar to the case of BSS, in which small subthreshold responses cannot be reliably deconvolved from the LFP. If all small non-spiking responses would be taken into account, the distribution of the relative proportion of active fibers decays exponentially with an excess of 1 mf connections. By excluding “undetectable” responses, the large-scale model generates proportions of granule cells activation similar to those obtained by BSS *in vitro* and *in vivo*. Note also that the case of 5 mossy fibers was not considered in BSS.

**
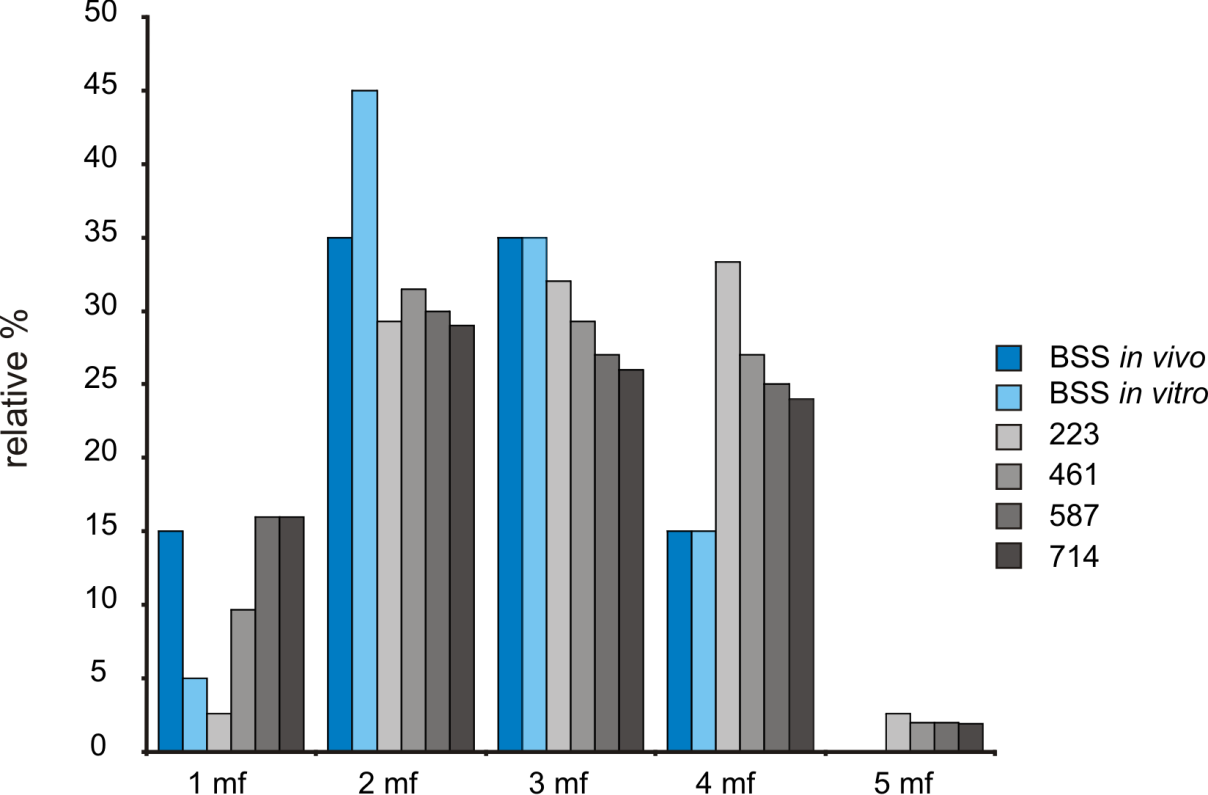
**

**Appendix S1 Figure 3. The impact of release probability in the large-scale model. (A)** The number of granule cells in a cluster scales with the number of mossy fibers (black line). The proportions of active granule cells scales with release probability (*p*) but is rather insensitive to cluster size (red lines). **(B)** The density of active granule cells decreases with distance from the core of the cluster. Profiles are reported at different *p* and for different numbers of mossy fibers. **(C)** Spikegrams at different *p* and for different numbers of mossy fibers. Red dots are first spikes and black dots second spikes in the same granule cells.

**
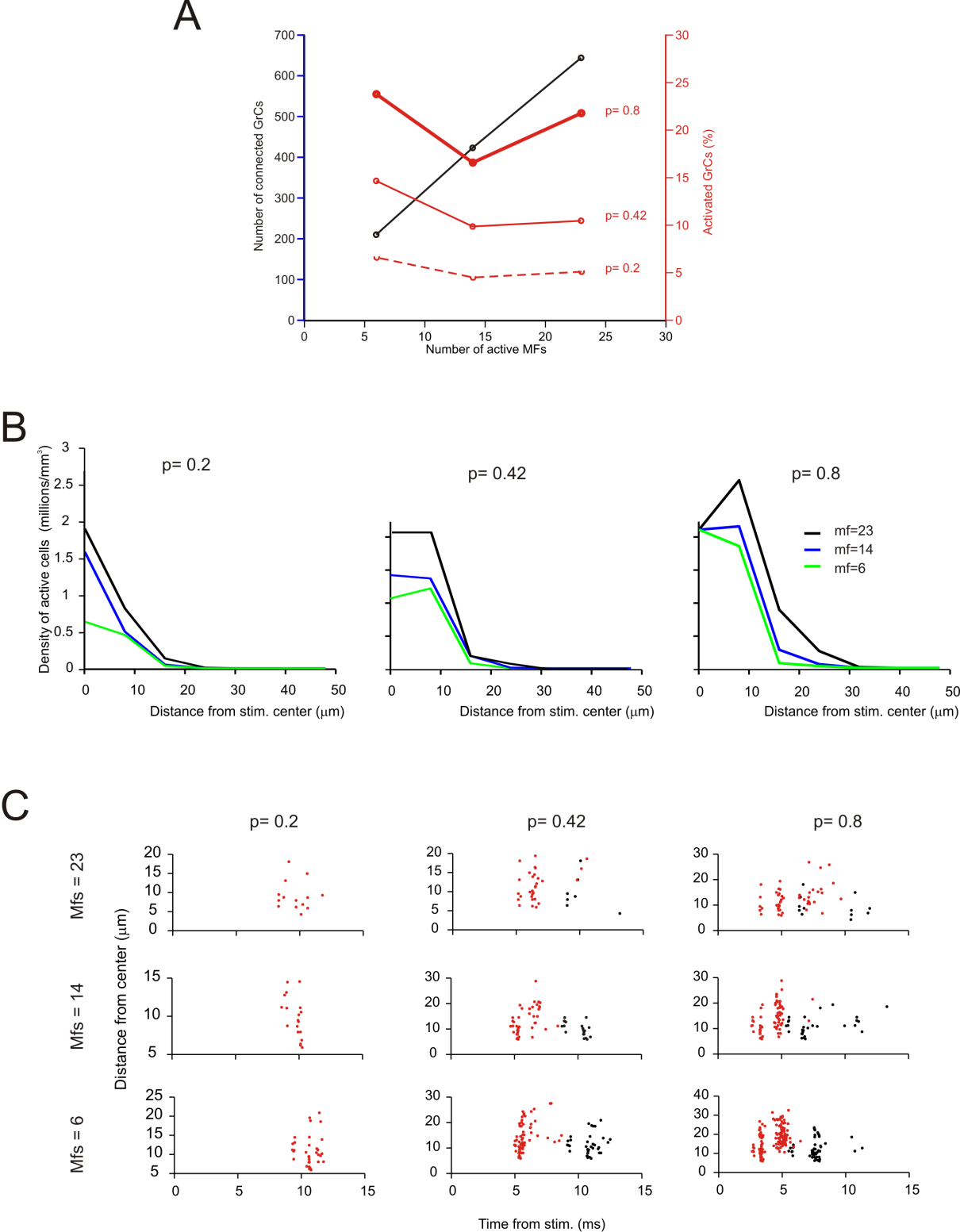
**

**REFERENCES**

1. Solinas S, Nieus T, D'Angelo E (2010) A realistic large-scale model of the cerebellum granular layer predicts circuit spatio-temporal filtering properties. Front Cell Neurosci 4: 12.

2. Kanichay RT, Silver RA (2008) Synaptic and cellular properties of the feedforward inhibitory circuit within the input layer of the cerebellar cortex. J Neurosci. United States. pp. 8955-8967.

3. Mapelli L, Rossi P, Nieus T, E. DA (2009) Tonic activation of GABAB receptors reduces release probability at inhibitory connections in the cerebellar glomerulus. J Neurophysiol 101: 3089-3099.

4. Sultan F, Heck D (2003) Detection of sequences in the cerebellar cortex: numerical estimate of the possible number of tidal-wave inducing sequences represented. J Physiol Paris 97: 591-600.

5. Mapelli J, D'Angelo E (2007) The spatial organization of long-term synaptic plasticity at the input stage of cerebellum. J Neurosci 27: 1285-1296.

6. Mapelli J, Gandolfi D, D'Angelo E (2010) Combinatorial responses controlled by synaptic inhibition in the cerebellum granular layer. J Neurophysiol 103: 250-261.

7. Mapelli J, Gandolfi D, D'Angelo E (2010) High-Pass Filtering and Dynamic Gain Regulation Enhance Vertical Bursts Transmission along the Mossy Fiber Pathway of Cerebellum. Front Cell Neurosci 4: 14.

8. D'Angelo E, De Zeeuw CI (2009) Timing and plasticity in the cerebellum: focus on the granular layer. Trends Neurosci 32: 30-40.

9. Roggeri L, Rivieccio B, Rossi P, D'Angelo E (2008) Tactile stimulation evokes long-term synaptic plasticity in the granular layer of cerebellum. J Neurosci 28: 6354-6359.

10. D’Angelo E, Solinas S (2011) Realistic modeling of large-scale networks: spatio-temporal dynamics and long-term synaptic plasticity in the cerebellum. IWANN, paper 189, in press
